# Factors associated with eating behaviors in older adults from a socioecological model perspective

**DOI:** 10.1186/s12889-023-16651-2

**Published:** 2023-09-05

**Authors:** Xue Wang, Yibo Wu, Juanxia Miao, Keping Pu, Wai-Kit Ming, Shuang Zang

**Affiliations:** 1 Department of Community Nursing, School of Nursing, China Medical University, No.77 Puhe Road, Shenyang North New Area, Shenyang, Liaoning Province 110122 China; 2 School of Public Health, Peking University, No.38 Xueyuan Road, Haidian District, Beijing, 100191 China; 3 Department of Infectious Diseases and Public Health, Jockey Club College of Veterinary Medicine and Life Sciences, City University of Hong Kong, To Yuen Building, No.31 To Yuen Street, Hong Kong, 999077 China

**Keywords:** Eating behavior, Older adults, Socioecological model, China

## Abstract

**Background:**

The eating behaviors of older adults are associated with multiple factors. To promote older adults’ healthy diets, it is imperative to comprehensively study the factors associated with the eating behaviors of the aging population group. This study aimed to probe the associated factors of older adults’ eating behaviors from a socioecological model (SEM) perspective.

**Methods:**

In 2021, a cross-sectional survey was performed to recruit participants in China. The survey data were analyzed using a multivariate generalized linear model to identify the factors associated with eating behaviors in older adults. Standardized regression coefficients (β) and 95% confidence intervals (CIs) were estimated using a multivariate generalized linear model.

**Results:**

The survey contained 1147 valid older adult participants. Multivariate generalized linear model results showed that older adults with older age [aged 71–80 (β = -0.61), ≥ 81 (β = -1.12)], conscientiousness personality trait (β = -0.27), and higher family health levels (β = -0.23) were inclined to have better eating behaviors. The older adults with higher education levels [junior high school and high school (β = 1.03), junior college and above (β = 1.71)], higher general self-efficacy (β = 0.09), more severe depression symptoms (β = 0.24), and employment (β = 0.82) tended to have poorer eating behaviors.

**Conclusions:**

This study identified factors that are specifically associated with older adults’ eating behaviors from an SEM perspective. The comprehensive multiple-angle perspective consideration may be a valuable idea for studying healthy eating behaviors in older adults.

## Introduction

Globally the population is aging at an unprecedented rate. By 2050, the global population aged 60 and older will double from 1.4 billion in 2015 to 2.1 billion [1]. The World Health Organization proposed that it is very important to promote the health of the older adult population in response to aging [2]. The trend of population aging has increased people’s interest in promoting the constituents of healthy aging.

One of the fundamental aspects of healthy aging is healthy eating behaviors [3]. The findings of epidemiological studies have shown that maintaining healthy eating behaviors could reduce the incidence of morbidity, particularly associated with cognitive decline and metabolic disease, thus reducing the cost of healthcare [4, 5]. Additionally, some studies have also indicated that healthy eating behaviors are associated with better health and quality of life for older adults [6, 7]. However, older adults may eat unhealthily due to physical problems or decreased appetite [8], putting them at risk for malnutrition [9]. The effects of malnutrition can be severe on the health, well-being, and autonomy of older adults [10, 11]. Therefore, it is crucial to identify the associated factors of older adults’ eating behaviors, with the aim of developing specific health promotion strategies tailored to the needs of this targeted group.

Several studies have examined the associated factors of older adults’ eating behaviors [12–14]. A study has revealed that older adults eating behaviors are associated with multiple factors, mainly at the individual level (e.g., income) [15]. Several other studies highlighted the role of social factors in older adults’ eating behaviors, such as social relationships and social support [16, 17]. In addition, older adults eating habits have also been demonstrated to be associated with family factors, for example, whether living alone [18, 19]. However, it should be recognized that most existing studies on factors associated with eating behavior in older adults tended to focus on only one or a few angles (e.g., individual level and social level), a holistic view of factors associated with older adults eating behavior is lacking, thus, a comprehensive framework should be established to sort out associated factors. Additionally, based on the bio-psycho-social medical model, the person is regarded as a whole and is affected by environmental, physiological, psychological, and social factors, it is very necessary to study the associated factors of eating behavior from a holistic point of view. Thus, this study introduced the socioecological model (SEM) to assist in an understanding of factors associated with older adults eating behaviors at multiple levels [20].

SEM is a useful tool for addressing health behaviors by the attribution of health outcomes to factors. SEM can provide a theoretical framework for understanding the interactions between individual and environmental factors that affect health outcomes and behaviors [21]. In this multilevel model, individual characteristics, individual behaviors, interpersonal networks, community, and policy levels are all considered factors that relate to health outcomes and behaviors and highlight the importance of taking into consideration these factors [22]. SEM has been widely applied in the study of health behaviors. For instance, Wang et al. [23] employed SEM to investigate the associated factors with willingness of using mobile health devices and obtained some comprehensive insight into these factors. A study by Zhang et al. [24] examined the factors associated with the utilization of online medical services based on SEM, and valuable insights were also drawn.

Therefore, the objective of this study is to evaluate multilevel factors associated with elderly eating behaviors from the SEM perspective.

## Methods

### Survey design and participants

From 10 to 2021 to 15 September 2021, we conducted a survey using a multistage sampling method from 31 (91% of the total) provinces/autonomous regions/municipalities in mainland China. The random number table method was used to select 120 cities, including the capital and two to six prefectural cities in each province and autonomous region. Based on the Chinese population pyramid, quota sampling was performed on the selected residents from 120 cities (the quota attributes are sex, age, and urban-rural distribution), ensuring the above variables’ distribution of the obtained samples was basically in line with the population feature. Participants were surveyed via the Wenjuanxing platform by investigators issuing participants one-on-one questionnaires. Participants who were able to think but were not sufficiently actionable to fill in the questionnaire were interviewed one-on-one by the investigators and provided assistance without intervention.

All participants provided informed consent before data collection, and all data were kept strictly confidential. This study obtained ethics approval from the Ethics Committee of Jinan University (No. JNUKY-2021-018). All methods in our study were performed following the guidelines and regulations of the Declaration of Helsinki.

### Sample size estimation

We calculated the sample size for a cross-sectional survey. The formula (N = Z_α_^2^ × p × q/(d^2^) [25]) estimated a sample size of 1068. The α of this formula represented the significance level, Z_α_ represented the statistic of the significance test, p × q represented the maximum estimation of variance, and d represented the permissible error.

#### Survey instruments

**Fig. 1:**
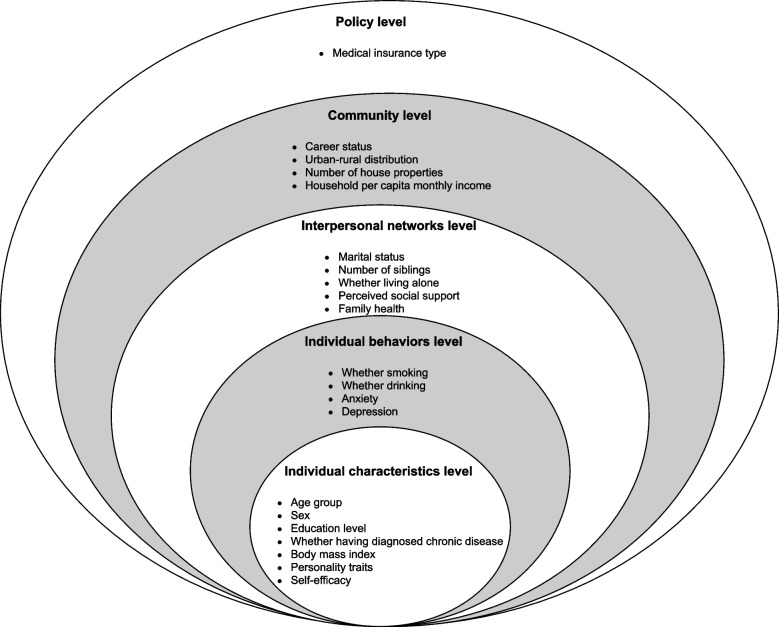
Factors associated with eating behaviors assembled based on the social-ecological model

The study questionnaires were composed of self-designed questionnaires and a series of standardized questionnaires focusing on the factors associated with older adults’ eating behaviors (Fig. 1).

#### Self-designed questionnaires

The self-designed questionnaires were a combination of general demographic characteristics (i.e., age, sex, education level, diagnosis of chronic diseases, body mass index (BMI), smoking and drinking status, marital status, living alone, career status, urban-rural distribution, and medical insurance type) and some basic family information (i.e., number of siblings, number of house properties, and household per capita monthly income).

### Standardized questionnaires

#### Eating behavior

The Eating Behavior Scale-Short Form (EBS-SF) was extracted from the 30-item Sakata Eating Behavior Scale (EBS) to evaluate participants’ dietary behaviors [26] and had good detection performance [27]. The scale consists of 7 items, including eating rhythm abnormalities, the feeling of satiety, eating habits, cognition of constitution, meal content, substitute eating and drinking, and motivation to eat. Each item is scored on a 4-point Likert scale, rated from 1 (strongly disagree) to 4 (strongly agree). Total summed EBS-SF scores range from 7 to 28, with higher scores reflecting worse eating behaviors. In this study, Cronbach’s α of the EBS-SF was 0.885.

#### Personality traits

The Big Five Inventory-10 (BFI-10) is used to evaluate participants’ personality traits [28]. There are five dimensions of personality in the BFI-10: extraversion, agreeableness, conscientiousness, neuroticism, and openness. Each personality dimension is measured by two items. And each item is scored on a 5-point Likert scale, rated from 1 (totally disagree) to 5 (totally agree). Reverse questions are scored from 1 (totally agree) to 5 (totally disagree). Higher individual dimension scores reflect greater levels of personality traits. As only two items per dimension are present in the BFI-10, no Cronbach’s α was calculated [29].

#### Self-efficacy

The New General Self-Efficacy Scale (NGSES) is used to evaluate participants’ perceived self-efficacy [30]. Each item is scored on a 5-point Likert scale, rated from 1 (strongly disagree) to 5 (strongly agree). Total summed NGSES scores range from 8 to 40, with higher scores reflecting greater self-efficacy. In this study, Cronbach’s α of the NGSES was 0.936.

#### Anxiety

The Generalized Anxiety Disorder-7 (GAD-7) is used to evaluate participants’ generalized anxiety symptoms [31]. Each item is scored on a 4-point Likert scale, rated from 0 (never) to 3 (nearly every day). Total summed GAD-7 scores range from 0 to 21, with higher scores reflecting more severe levels of anxiety symptoms. In this study, Cronbach’s α of the GAD-7 was 0.951.

#### Depression

The Patient Health Questionnaire-9 (PHQ-9) is used to evaluate participants’ depression symptoms [32]. Each item is scored on a 4-point Likert scale, rated from 0 (never) to 3 (nearly every day). Total summed PHQ-9 scores range from 0 to 27, with higher scores reflecting more severe levels of depression symptoms. In this study, Cronbach’s α of the PHQ-9 was 0.930.

#### Perceived social support

The Perceived Social Support Scale (PSSS) is used to evaluate participants’ perceived social support from family, friends, and significant others [33]. Each item is scored on a 7-point Likert scale, rated from 1 (strongly disagree) to 7 (strongly agree). Total summed PSSS scores range from 12 to 84, with higher scores reflecting a higher level of perceived social support. In this study, Cronbach’s α of the PSSS was 0.952.

### Family health

The Family Health Scale-Short Form (FHS-SF) is used to evaluate participants’ family health environments [34]. Each item is scored on a 5-point Likert scale, rated from 1 (strongly disagree) to 5 (strongly agree). Reverse questions are reverse-scored. Total summed FHS-SF scores range from 10 to 50, with higher scores reflecting a higher level of family health. In this study, Cronbach’s α of the FHS-SF was 0.848.

### Statistical methods

First, the Kolmogorov–Smirnov test was conducted to examine whether continuous variables followed a normal distribution. Continuous variables had a nonnormal distribution and were displayed as the median [interquartile range (IQR)]. Categorical variables were displayed as numbers (percentages). Second, the associations between the study variables and eating behaviors were measured using a univariate generalized linear model. Third, we examined the potential multicollinearity issue by calculating the variance inflation factor (VIF). The multicollinearity test demonstrated no collinearity among the independent variables in this study (maximum VIF = 3.5). Fourth, significant variables from the univariate generalized linear models (with *P* < 0.05) were investigated in multivariable generalized linear models. All two-sided *P* values < 0.05 were considered statistically significant. All statistical analyses were conducted using SPSS 19.0 (SPSS Inc., Chicago, IL, USA).

## Results

### Participant characteristics

A total of 11,031 questionnaires were initially collected, and after excluding 9884 questionnaire participants aged < 60 years old, the remaining 1147 questionnaires were included. In this study, 50.65% of participants were male, 61.90% had been diagnosed with chronic disease, 12.82% lived alone, and 42.98% lived in rural areas. The median score for the participants’ eating behavior scale was 14 points, self-efficacy was 28 points, anxiety was 2 points, depression was 4 points, perceived social support was 60 points, and family health was 38 points (Table 1).

**Table 1:** Participants characteristics based on the social-ecological model (*n* = 1147)

Variables	Value
***Individual characteristics level***	
Age group (year), n (%)	
60–70	514 (44.81%)
71–80	525 (45.77%)
≥ 81	108 (9.42%)
Sex, n (%)	
Male	581 (50.65%)
Female	566 (49.35%)
Education level, n (%)	
Primary school and below	506 (44.12%)
Junior high school and high school	431 (37.58%)
Junior college and above	210 (18.31%)
Whether having diagnosed chronic disease, n (%)	
No	437 (38.10%)
Yes	710 (61.90%)
BMI kg/m^2^, median (IQR)	22.00 (19.90–24.20)
Personality traits (scores), median (IQR)	
Extraversion	6.00 (5.00–7.00)
Agreeableness	7.00 (6.00–8.00)
Conscientiousness	7.00 (6.00–8.00)
Neuroticism	6.00 (5.00–6.00)
Openness	6.00 (5.00–7.00)
Self-efficacy (scores), median (IQR)	28.00 (24.00–32.00)
***Individual behaviors level***	
Whether smoking, n (%)	
Nonsmoker	753 (65.65%)
Ex-smoker	229 (19.97%)
Smoker	165 (14.39%)
Whether drinking, n (%)	
No	781 (68.09%)
Drank before 30 days	118 (10.29%)
Drank in 30 days	248 (21.62%)
Anxiety (scores), median (IQR)	2.00 (0–7.00)
Depression (scores), median (IQR)	4.00 (1.00–8.00)
***Interpersonal networks level***	
Marital status, n (%)	
Have no partner	253 (22.06%)
Have a partner	894 (77.94%)
Number of siblings, n (%)	
≤ 1	357 (31.12%)
2	403 (35.14%)
≥ 3	387 (33.74%)
Whether living alone, n (%)	
No	1000 (87.18%)
Yes	147 (12.82%)
Perceived social support (scores), median (IQR)	60.00 (51.00–71.00)
Family health (scores), median (IQR)	38.00 (34.00–43.00)
***Community level***	
Career status, n (%)	
Retired	647 (56.41%)
Employed	500 (43.59%)
Urban‒rural distribution, n (%)	
Rural	493 (42.98%)
Urban	654 (57.02%)
Number of house properties, n (%)	
0	90 (7.85%)
1	759 (66.17%)
≥ 2	298 (25.98%)
Household per capita monthly income (yuan), n (%)	
≤ 3000	471 (41.06%)
3001–6000	406 (35.40%)
≥ 6001	270 (23.54%)
***Policy level***	
Medical insurance type, n (%)	
Self-pay	130 (11.33%)
Resident basic medical insurance	771 (67.22%)
Employee basic medical insurance	207 (18.05%)
Commercial insurance and socialized medicine	39 (3.40%)
EBS-SF scores, median (IQR)	14.00 (11.00–17.00)

Note: Total percentages within categories may not equal 100% due to rounding

*Abbreviations*: *IQR *Interquartile range, *BMI *Body mass index

### Association between study variables and eating behaviors

The univariate generalized linear model showed that most variables in this study were associated with eating behaviors (*P* < 0.05). For example, individuals with older age [aged 71–80 (β = -1.19), ≥ 81 (β = -1.15)], extraversion (β = -0.19), agreeableness (β = -0.79), conscientiousness personality trait (β = -0.91) were more likely to have healthy eating behaviors. Individuals with higher education levels [junior high school and high school (β = 0.84), junior college and above (β = 1.80)], neuroticism personality trait (β = 0.62), higher levels of anxiety (β = 0.36) and depression (β = 0.35) were likely to have unhealthy eating behaviors (Table 2). The results from the multivariate generalized linear model indicated that participants with older age [aged 71–80 (β = -0.61), ≥ 81 (β = -1.12)], conscientiousness personality trait of BFI-10 (β = -0.27), and higher family health levels (β = -0.23) were inclined to have better eating behaviors. The participants with higher education levels [junior high school and high school (β = 1.03), junior college and above (β = 1.71)], had higher general self-efficacy (β = 0.09), had more severe depression symptoms (β = 0.24), and were employed (β = 0.82) tended to have worse eating behaviors (Table 3).

**Table 2:** Univariate generalized linear model analysis of associations between study variables and eating behaviors (*n* = 1147)

Variables	*β* (95% CI)	*P*
***Individual characteristics level***		
Age group (Ref: 60–70)		
71–80	-1.19 (-1.76–-0.62)	< 0.001
≥ 81	-1.15 (-2.12–-0.18)	0.020
Sex (Ref: Male)		
Female	-0.10 (-0.65-0.44)	0.706
Education level (Ref: Primary school and below)		
Junior high school and high school	0.84 (0.24–1.43)	0.006
Junior college and above	1.80 (1.05–2.55)	< 0.001
Whether having diagnosed chronic disease (Ref: No)		
Yes	-0.90 (-1.46–-0.34)	0.002
BMI	0.03 (-0.06-0.11)	0.549
Personality traits		
Extraversion	-0.19 (-0.37–-0.01)	0.035
Agreeableness	-0.79 (-0.97–-0.62)	< 0.001
Conscientiousness	-0.91 (-1.08–-0.74)	< 0.001
Neuroticism	0.62 (0.43–0.81)	< 0.001
Openness	0.07 (-0.11-0.26)	0.446
Self-efficacy	-0.07 (-0.12–-0.02)	0.008
***Individual behaviors level***		
Whether smoking (Ref: Nonsmoker)		
Ex-smoker	-0.64 (-1.34-0.05)	0.071
Smoker	0.41 (-0.38-1.21)	0.306
Whether drinking (Ref: No)		
Drank before 30 days	-0.26 (-1.17-0.65)	0.577
Drank in 30 days	0.89 (0.22–1.56)	0.009
Anxiety	0.36 (0.30–0.42)	< 0.001
Depression	0.35 (0.30–0.40)	< 0.001
***Interpersonal networks level***		
Marital status (Ref: Have no partner)		
Have a partner	0.07 (-0.59-0.72)	0.846
Number of siblings (Ref: ≤1)		
2	-0.68 (-1.35–-0.02)	0.044
≥ 3	-1.36 (-2.03–-0.69)	< 0.001
Whether living alone (Ref: No)		
Yes	0.96 (0.14–1.77)	0.022
Perceived social support	-0.08 (-0.10–-0.06)	< 0.001
Family health	-0.30 (-0.34–-0.26)	< 0.001
***Community level***		
Career status (Ref: Retired)		
Employed	0.63 (0.08–1.18)	0.024
Urban‒rural distribution (Ref: Rural)		
Urban	0.38 (-0.18-0.93)	0.182
Number of house properties (Ref: 0)		
1	-1.30 (-2.32–-0.27)	0.014
≥ 2	-1.18 (-2.29–-0.08)	0.037
Household per capita monthly income (Ref: ≤3000)		
3001–6000	0.44 (-0.19-1.06)	0.172
≥ 6001	0.86 (0.16–1.57)	0.016
***Policy level***		
Medical insurance type (Ref: Self-pay)		
Resident basic medical insurance	-2.30 (-3.16–-1.44)	< 0.001
Employee basic medical insurance	-1.98 (-3.00–-0.97)	< 0.001
Commercial insurance and socialized medicine	1.09 (-0.57-2.75)	0.197

Note: Reference means a category in which all reference variables take the value of zero

*Abbreviations*: *β *regression coefficients, *CI *Confidence interval, *Ref *Reference, *BMI *Body mass index

**Table 3:** Multivariate generalized linear model analysis of associations between study variables and eating behaviors (*n* = 1147)

Variables	*β (*95%CI)	*P*
***Individual characteristics level***		
Age group (Ref: 60–70)		
71–80	-0.61 (-1.12–-0.11)	0.017
≥ 81	-1.12 (-1.99–-0.26)	0.011
Education level (Ref: Primary school and below)		
Junior high school and high school	1.03 (0.46–1.60)	< 0.001
Junior college and above	1.71 (0.95–2.47)	< 0.001
Personality traits		
Extraversion	0.04 (-0.12-0.20)	0.653
Agreeableness	-0.17 (-0.36-0.02)	0.086
Conscientiousness	-0.27 (-0.45–-0.08)	0.005
Neuroticism	0.16 (-0.02-0.35)	0.084
Self-efficacy	0.09 (0.04–0.15)	< 0.001
*** Individual behaviors level***		
Anxiety	-0.02 (-0.12-0.08)	0.717
Depression	0.24 (0.16–0.33)	< 0.001
***Interpersonal networks level***		
Number of siblings (Ref: ≤1)		
2	-0.15 (-0.74-0.44)	0.610
≥ 3	-0.39 (-1.03-0.25)	0.230
Whether living alone (Ref: No)		
Yes	0.18 (-0.53-0.89)	0.623
Perceived social support	0.02 (-0.01-0.05)	0.142
Family health	-0.23 (-0.28–-0.18)	< 0.001
***Community level***		
Career status (Ref: Retired)		
Employed	0.82 (0.29–1.35)	0.002
Household per capita monthly income (Ref: ≤3000)		
3001–6000	-0.49 (-1.38-0.41)	0.285
≥6001	-0.41 (-1.39-0.57)	0.412

Note: Reference means a category in which all reference variables take the value of zero

*Abbreviations*: *β *Regression coefficients, *CI *Confidence interval, *Ref *Reference

## Discussion

The current study examined the factors associated with eating behaviors in older adults based on SEM. The results showed that older age, higher educational level, conscientiousness personality trait, higher self-efficacy at the individual characteristics level; more severe depression symptoms at the individual behaviors level; higher family health at the interpersonal networks level; and employed career status at community level were associated with eating behaviors in older adults.

This study showed that SEM can help to better sort out and study the factors associated with older adults eating behaviors from a relatively comprehensive perspective. The results indicated that the factors associated with the eating behaviors of older adults are mainly concentrated in the SEM level of individual characteristics, and second, the level of individual behaviors, interpersonal networks, and community levels have also been found to have relevant factors. It is worth carrying out in-depth analysis and corresponding intervention guidance for improving older adults’ healthy eating behaviors from these perspectives in the future.

Our study revealed that older age was associated with healthy eating behaviors, which was in line with previous studies [35, 36]. The evidence suggested that aging was positively associated with healthy eating behaviors [37]. Older adults tend to maintain healthy eating behaviors, including consuming more fresh vegetables and fruits rather than high-fat foods [38]. With increasing age, older adults have gradually become aware of aging and the decline of digestive system functions [39], they may thus pay more attention to their health, such as health preservation, and diet. Additionally, in China, there is a high prevalence of digestive system illnesses in older adults, such as gastritis and colitis [40, 41], as well as a high prevalence of hypertension, diabetes, and hyperlipidemia [42, 43]. To comply with the need for treatment and care for digestive system diseases and other chronic diseases, many older adults tend to adopt healthy eating practices, such as eating soft food, low-salt and low-fat meals, having more meals a day but less food at each, etc. [6, 44]. Generally, older adults are likely to have more spare time after retirement, they thus have more time and energy to cook healthier foods, so they consume fewer unhealthy foods.

In this study, we found that the conscientiousness personality trait rated by BFI-10 was associated with more healthful eating behaviors, this finding was consistent with other studies among older adults [45, 46]. Individuals with high conscientiousness appear to maintain restrained regulatory eating and practice less counterregulatory emotional or external eating [47], and may be more capable of avoiding or controlling emotional stressors, resulting in decreased emotional eating and further decreased unhealthy eating [48]. Additionally, individuals with high conscientiousness may have more self-control resources to maintain their diet goals in tempting food situations and inhibit their eating enjoyment goals, thus adhering to a balanced and healthy diet [49]. As individuals generally pay more attention to their health in old age. Older adults should be encouraged to pay conscientious attention to diet. This highlights the need for personality traits that should be considered in the promotion of healthy eating among older adults.

The present study revealed an intriguing finding whereby a positive association was observed between a high education level and unhealthy eating behaviors among older adults. This finding contrasted with the expected trend observed in prior studies wherein individuals with higher education tend to exhibit healthier dietary habits [50, 51]. One potential explanation for this intriguing phenomenon lies in the possibility of overconfidence among individuals with elevated educational backgrounds when applying nutritional knowledge, resulting in suboptimal dietary choices. This phenomenon finds support in the research, which suggested that those with higher education levels might be more susceptible to specific nutritional information, potentially deviating from a comprehensive dietary approach [52]. Additionally, individuals with higher education may experience increased societal pressures, demanding a delicate balance between their professional, social, and familial responsibilities during their tenure [53]. These pressures could lead to decisions favoring expedient and convenient food choices, potentially at the expense of adhering to the principles of a wholesome diet [54]. This supposition resonates with the findings of Putri et al., which emphasized that individuals experiencing elevated stress levels tend to opt for high-calorie, low-nutrient foods [55]. These dietary habits tend to become ingrained from a young age and persist into later life, forming enduring patterns [56]. Furthermore, the interplay of social identity and cultural factors might wield influence over this observed association. Notably, individuals with advanced educational backgrounds may possess distinct social identities and cultural values that impact their dietary preferences [57]. Specific societal subgroups might exhibit a preference for traditional high-sugar, high-fat foods, thereby conflicting with the recommendations of a health-conscious diet [58]. The implications of this noteworthy revelation underscore the importance of addressing potential determinants contributing to unhealthy dietary behaviors among older adults with elevated educational status. Consequently, the development of targeted and effective public health interventions tailored to this specific demographic becomes imperative. Given the ongoing global aging phenomenon, promoting healthful dietary habits, and tackling the unique challenges faced by older adults with higher educational backgrounds emerge as essential endeavors for optimizing both their health and overall quality of life.

This study also confirmed that high self-efficacy was associated with worse eating behaviors among older adults. In contrast to prior research, which has suggested that older adults with high self-efficacy exhibit better coping abilities in challenging situations, display goal-oriented behaviors, and are more likely to adopt healthy eating habits due to the significance of overcoming failures and making persistent efforts to maintain healthy dietary patterns [59, 60]. The underlying reason for the observed association between high self-efficacy and worse eating behaviors among older adults could be attributed to the complexity of psychological factors associated with eating behaviors in this population. First, it is crucial to consider the impact of self-regulation abilities. Despite having high self-efficacy, individuals may face hindrances in regulating food intake due to factors like emotional eating tendencies or external food-related cues, resulting in self-efficacy not always translating into optimal eating behaviors [61, 62]. Second, the role of habit formation and automaticity in eating behaviors should not be overlooked. Prior research has highlighted that habits play a significant role in determining eating patterns, especially among older adults who may have established long-standing routines [63]. Consequently, even with high self-efficacy, individuals may experience overshadowed confidence in making consistently healthy choices if unhealthy eating habits have become ingrained over time [64]. Furthermore, the dietary decisions of older adults are frequently shaped by their social networks, familial interactions, and the accessibility of healthful food alternatives within their immediate surroundings [65]. As a result, even those individuals possessing elevated self-efficacy could yield to external influences or contextual limitations, potentially predisposing them towards favoring less nutritious dietary selections [66]. Therefore, while self-efficacy remains a crucial determinant, its association can be intricately interwoven with external factors, necessitating a comprehensive understanding of the multifaceted dynamics that underlie the dietary behaviors of older adults.

Our study discovered that higher family health levels were associated with healthy eating behaviors. It has been demonstrated that the family environment can have a significant impact on health behaviors [67]. Previous studies found that a positive family system can contribute to establishing and promoting beneficial health behaviors through the exemplary role, provision of healthy foods, and encouragement of healthy eating behaviors [68]. The Chinese government has also realized the necessity of improving the health of the whole population and proposed the Healthy China Plan in 2016, with the aim of promoting people’s health and then improving the level of national health literacy [69], which further improves the level of family health literacy to a certain extent. The family is the tiniest cell of society and plays a vital role in both individual life and society. This study found that the health of individuals cannot be separated from the health of the family, and it is very important to implement the guidance of the national health environment into specific family life. It is thus clear that policymakers who promote healthy eating behaviors should give full play to the positive role of family health resources in establishing and promoting good eating behaviors.

We also found that more severe depression symptoms were associated with worse eating behaviors, which was consistent with prior research [70, 71]. Individuals with depression symptoms are more likely to increase unhealthy food consumption [72] and have unhealthy eating habits [73]. Furthermore, depression affects eating motivations and drives food choices toward less healthy foods [74]. There was, however, approximately 40% of older adults aged 60 or older in China who reported having depression symptoms [75]. It is therefore necessary to focus on depressed older adults. While considering addressing the psychological problems of older adults, lifestyle problems such as diet should also be considered to promote the improvement of their overall health.

Moreover, our study revealed that the career status of employed was associated with unhealthy eating behaviors. Employed older adults may put in more physical and mental effort to complete the work, which is very challenging for physical strength, time, and energy [76]. Therefore, older adults in employment can crowd out some of the time that could be divided between food preparation, learning about dietary management, etc. Additionally, numerous studies have confirmed that work-related stress is associated with unhealthy eating behaviors [77, 78]. Compared to other working-age populations, older adults are at significantly higher risk of stress because of their diminished physical and mental abilities [79, 80]. Therefore, it is necessary to pay attention to the dietary problems of employed older adults.

Several potential limitations of this study should be acknowledged. First, due to the cross-sectional study design, we cannot infer causal implications. Second, there is the possibility that data may be to some extent biased since some information was self-reported. Third, we could not exclude participants who were on a weight-loss diet, so careful consideration should be given to the possibility of measurement bias, as well as continued evaluation of the EBS-SF. Fourth, although some variables (i.e., age, sex, and urban-rural distribution) had been quota, there may have been some uneven data distribution (e.g., education level, smoking, and drinking), which could have a certain effect on the outcomes. Fifth, the data collection in this study was during the COVID-19 epidemic and sometimes was subject to epidemic containment management, which may affect eating behaviors. Sixth, although we have considered multiple factors related to eating behaviors in older adults from a socioecological model perspective, this study lacks the inclusion of nutritional status and cognitive functioning as variables. Nutritional status is significantly associated with eating behaviors in older adults, while cognitive functioning also plays a crucial role in their dietary decision-making and habits. Therefore, the omission of these variables may have impacted a comprehensive understanding of eating behaviors in older adults. Future research should incorporate measures of nutritional status and cognitive functioning to gain a more comprehensive insight into the factors associated with eating behaviors among older adults.

## Conclusions

This study identified factors associated with older adults eating behaviors and provided an in-depth understanding of these factors at five levels based on SEM. The findings provide information about integral assessments of older adults’ eating behaviors and may contribute to the development of targeted and multipronged health-promoting strategies to improve the health of older adults. Additionally, strategies that promote healthier eating behaviors in older adults from a holistic perspective may contribute to healthy aging through better nutrition.

## Data Availability

The datasets used and/or analyzed during the current study are available from the corresponding author upon reasonable request.
